# Predictors of clinical outcomes after cardiac resynchronization therapy in patients ≥75 years of age: a retrospective cohort study

**DOI:** 10.1186/s12877-019-1351-4

**Published:** 2019-11-21

**Authors:** Laure Champ-Rigot, Anne-Laure Cornille, Pierre Ollitrault, Arnaud Pellissier, Mathieu Chequel, Damien Legallois, Paul Milliez

**Affiliations:** 1 Normandie University, UNICAEN, CHU de Caen Normandie, Service de Cardiologie, EA4650 (Signalisation, électrophysiologie et imagerie des lésions d’ischémie-reperfusion myocardique), 14000 Caen, France; 2 Normandie University, UNICAEN, CHU de Caen Normandie, Service de Cardiologie, 14000 Caen, France

**Keywords:** Resynchronization therapy, Heart failure, Aged, Treatment outcome

## Abstract

**Background:**

Cardiac resynchronization therapy has been shown to benefit selected patients with heart failure and reduced ejection fraction. Older patients have been underrepresented in randomized trials. This study was conducted to determine whether predictive factors for cardiac resynchronization therapy outcomes differ in patients older and younger than 75 years of age.

**Methods:**

Consecutive patients who received a cardiac resynchronization device cardiac resynchronization therapy between 2013 and 2016 in our center were retrospectively included in this cohort study. The primary endpoint was cardiac resynchronization therapy effectiveness, which was defined as survival for one year with both no heart failure hospitalization and improvement by one or more NYHA class. The secondary endpoints were mortality, complications, and device therapies.

**Results:**

Among the 243 patients included, 102 were ≥ 75 years old. Cardiac resynchronization therapy effectiveness was observed in 70 patients (50%) < 75 years old and in 48 patients (47%) ≥75 years old (*p* = 0.69). NYHA class ≥III (OR = 6.02; CI95% [1.33–18.77], *p* = 0.002) was a predictive factor for cardiac resynchronization therapy effectiveness only in the ≥75-year-old group, while atrial fibrillation was independently negatively associated with the primary endpoint in the < 75-year-old group (OR = 0.28; CI95% [0.13–0.62], *p* = 0.001). The one-year mortality rate was 14%, with no difference between age groups. Rescue cardiac resynchronization therapy and atrial fibrillation were independent predictive factors for mortality in both age groups. Eighty-two complications occurred in 45 patients (19%), with no difference between groups. Defibrillator use and QRS duration were independent predictive factors for complications in both age groups. There was no difference between groups considering device therapies.

**Conclusion:**

At one year, cardiac resynchronization therapy response is not compromised by patient age. In older patients, highly symptomatic individuals with NYHA class ≥III have better outcomes after cardiac resynchronization therapy.

## Background

Cardiac resynchronization therapy (CRT) has become a standard therapy for patients with chronic heart failure (HF) related to reduced left ventricular ejection fraction (LVEF). Large clinical trials have demonstrated CRT benefits on symptoms, quality of life, as well as morbidity and mortality reduction [1–4]. The current guidelines recommend CRT for selected patients regardless of age status [5], but older patients, defined as individuals aged ≥65, especially those aged ≥75, have been underrepresented in these studies [6]. The prevalence of HF increases with age, and the European Survey on CRT reported that the median age of patients with HF was 70, with 31% of the patients being aged ≥75 [7] . A few reports have previously highlighted the feasibility and efficiency of CRT in older patients, but little is known about the predictors of clinical responses in this population [8–12].

We aimed to determine age-related predictive factors for clinical outcomes in older patients receiving CRT.

## Methods

We designed a retrospective cohort study according to the guidelines for reporting observational studies and fulfilling the STROBE statement items, detailed in Additional file 1 [13].

### Study population

We retrospectively included all consecutive patients referred to Caen Normandy University Hospital for CRT device implantation between January 2013 and December 2016. CRT devices were implanted according to the most recent European guidelines [14]: New York Heart Association (NYHA) functional class II to ambulatory IV, LVEF ≤35%, QRS duration ≥120 ms and left-bundle branch block (LBBB) QRS morphology or QRS duration ≥150 ms and non-LBBB QRS morphology, despite optimized medical treatment. After publication of the latest guidelines in 2016 [5], the minimum QRS duration required was 130 ms in cases of LBBB QRS morphology. Patients with a ventricular pacing indication and reduced LVEF as well as those who had a previous device upgraded to a CRT pacemaker or CRT defibrillator were also included. We also considered patients who underwent implantation for rescue CRT, which is defined as the implantation of a CRT device for amine-dependent end-stage HF. We excluded patients under 18 years of age and those who were lost to follow-up. We defined older patients as those who were ≥ 75 years old at the time of implantation and then divided our population into two age groups: individuals aged < 75 and individuals aged ≥75.

### Device therapy

The CRT device was implanted using standard techniques. In patients with atrial fibrillation (AF), atrioventricular node ablation was performed when medical treatment failed to control their heart rate. The choice between a defibrillator and pacemaker for the primary prevention of sudden cardiac death, as well as baseline programming, was at the discretion of the attending physician.

### Data collection

Evaluation of the candidates for CRT implantation, except those undergoing rescue-CRT, had to be performed in our center within three months before the procedure, and included a 12-lead electrocardiogram and a standard two-dimensional echocardiogram performed with the IE 33™ or Epic 5™ system (Philips Healthcare, Amsterdam, Netherlands) with the measurement of LVEF using Simpson’s biplane method. Clinical, biological, electrocardiogram and echocardiographic data were anonymously collected.

### Follow-up and clinical endpoints

Patients’ follow-ups were scheduled according to our standard of care with a physical examination and device interrogation before hospital discharge, at one month, at six months and at one year. A remote monitoring system was proposed if appropriate.

The primary endpoint was CRT effectiveness, defined as a modified combined clinical score, which has been previously described as follows [9]: survival for one year with no heart failure hospitalization and improvement by ≥ one NYHA class. The secondary endpoints were all-cause mortality, complications, and the occurrence of device therapies in patients with a defibrillator. Appropriate device therapy was defined as anti-tachycardia pacing and/or internal shocks delivered to terminate sustained ventricular arrhythmia. Device therapy delivered in any other circumstance was considered inappropriate.

### Statistical analysis

Categorical variables were expressed as numbers and percentages and compared using Pearson’s chi-squared test or Fisher’s exact test depending on whether the data met the criteria for a given test. Continuous variables were expressed as the mean and standard deviation if the data were normally distributed or the median and interquartile range if the data were not normally distributed. The data were then compared using Student’s t-test or the Mann-Whitney U test. The association between the baseline characteristics and the occurrence of clinical events was evaluated by univariate analysis. Variables with *p* values ≤0.20 in univariate analysis were then introduced in multivariate analysis using a binary logistic regression model with Wald’s step-by-step method. The survival time for the primary endpoint was defined as the number of days between implantation and the first event. The Kaplan-Meier method was used to construct survival curves, and the log-rank test used to conduct comparisons among groups. Statistical significance was set at a two-tailed probability level of < 0.05. All analyses were performed using IBM SPSS Statistics for Windows version 20.0 (IBM Corp. Released 2011. Armonk, NY: IBM Corp.).

## Results

### Baseline characteristics

Two hundred fifty patients underwent CRT implantation; among them, 7 were excluded because of missing follow-up data. Hence, our study population comprised the remaining 243 patients, of which 102 (42%) were ≥ 75 years old. The baseline characteristics according to the age groups are summarized in Table 1. The patients were predominantly male, but there were more females in the ≥75-year-old group (23%) than in the < 75-year-old group (13%) (*p* = 0.04). A larger proportion of the patients ≥75 years old compared with those ≥75 years old had severe symptoms according to the NYHA class, had chronic kidney disease (CKD, defined by estimated glomerular filtration < 60 ml/min/1.73 m^2^), and were receiving loop diuretics, but fewer patients in this group were receiving aldosterone antagonists. However, patients ≥75 years old were less likely to have defibrillator than those < 75 years old (52% versus 87%, respectively; *p* <  0.001). Both groups had a comparable level of LVEF impairment, with a median LVEF of 28% (23.5–30), and mostly a LBBB QRS morphology (79%). There was no difference regarding the proportion of upgraded devices, CRT for rescue therapy and time of the procedure.

**Table 1:** Baseline characteristics and outcomes

	< 75 years old (*n* = 141)	≥75 years old (*n* = 102)	*p* value
Age (years)	64 (10)	79 (7)	< 0.001
Male sex, n (%)	122 (87)	78 (77)	0.04
Ischemic cardiomyopathy, n (%)	71 (50)	63 (62)	0.08
Atrial fibrillation, n (%)	66 (47)	56 (57)	0.14
Diabetes mellitus, n (%)	44 (31)	29 (29)	0.71
CKD, n (%)	62 (49)	68 (72)	0.001
NYHA functional class, n (%)			
I	3 (2)	1 (1)	0.01
II	57 (40)	22 (21)	
III	52 (37)	62 (61)	
IV ambulatory	7 (5)	7 (7)	
IV in hospital	22 (16)	10 (10)	
Rescue CRT, n (%)	14 (10)	8 (8)	0.58
Beta blocker, n (%)	119 (85)	78 (79)	0.21
ACEI or ARB, n (%)	112 (80)	71 (72)	0.14
Aldosterone antagonist, n (%)	71 (51)	36 (36)	0.03
Loop diuretic, n (%)	105 (75)	85 (86)	0.04
Ivabradine, n (%)	21 (15)	11 (11)	0.38
Anticoagulation therapy, n (%)	73 (52)	59 (58)	0.33
Anti-platelet agent, n (%)	73 (52)	55 (54)	0.60
QRS morphology, n (%)			
LBBB	110 (78)	82 (80)	0.50
RBBB	10 (7)	6 (6)	
NIVCD	3 (2)	0	
Paced QRS	18 (13)	14 (14)	
LVEF (%)	28 (6)	28 (7)	0.47
CRT-D, n (%)	122 (87)	53 (52)	< 0,001
Upgrade, n (%)	41 (29)	25 (25)	0.43
Effectiveness, n (%)	70 (50)	48 (47)	0.69
One-year survival	124 (88)	85 (83)	0.31
NYHA improvement	86 (61)	67 (66)	0.72
No admission for HF	107 (76)	71 (70)	0.33
Complications, n in n patients (%)	55 in 30 (21)	27 in 15 (15)	0.19
Reintervention, n (% of complications)	24 (44)	9 (33)	0.07
Lead displacement, n (% of complications)	19 (34)	7 (26)	0.10
Implantation failure, n (% of complications)	3 (5)	5 (19)	0.23
Infection, n (% of complications)	6 (11)	3 (11)	0.59
Pneumothorax, n (% of complications)	2 (4)	2 (7)	0.74
Perforating lead, n (% of complications)	0	1 (4)	0.24
Pericardial effusion, n (% of complications)	1 (2)	0	0.39
Hematoma, n (% of complications)	0	0	NA

Continuous variables are reported as medians and interquartile ranges; categorical variables are reported as numbers and percentages. ACEI: angiotensin-converting enzyme inhibitor; ARB: angiotensin II receptor blocker; CKD: chronic kidney disease (defined by estimated glomerular filtration < 60 ml/mn/1.73 m^2^); CRT: cardiac resynchronization therapy; CRT-D: CRT with defibrillator; HF: heart failure; LBBB: left bundle branch block; LVEF: left ventricular ejection fraction; NYHA: New York Heart Association; NIVCD: nonspecific intraventricular conduction delay; RBBB: right bundle branch block

### Primary endpoint

There was no difference in CRT effectiveness between age groups; 70 patients were considered responders out of the 141 (50%) in the < 75-year-old group, whereas 48 patients out of the 102 (47%) in the ≥75-year-old group were considered responders (*p* = 0.69). The outcomes are detailed in Table 1. In univariate and multivariate analyses, NYHA class ≥III before implantation was associated with the CRT response in the overall population (OR = 3.30; 95% confidence interval (CI95%) [1.70–6.51], *p* <  0.001). NYHA class ≥III was strongly predictive of CRT effectiveness among older patients (OR = 6.02; CI95% [1.33–18.77], *p* = 0.002). In the < 75-year-old group, there was a trend towards a better CRT response in patients with a previous NYHA class ≥III (*p* = 0.07). In univariate analysis, AF, CKD and rescue CRT were negatively associated with the CRT response. Only AF and CKD remained predictors of CRT nonresponse in the multivariate analysis of the overall population. Moreover, the AF negative impact was significant only in the < 75-year-old group (OR = 0.28; CI95% [0.13–0.62], *p* = 0.001) and was not significant in the ≥75-year-old group. Defibrillator use was not associated with the primary endpoint. The results of the univariate and multivariate analyses in the ≥75-year-old group are listed in Table 2 and those in the < 75-year-old group are provided in Table 3.

**Table 2:** Univariate and multivariate analyses of outcomes in the ≥75-year-old group

	Univariate analysis		Multivariate analysis (model 1 with atrial fibrillation)		Multivariate analysis (model 2 with anticoagulation)	
	OR (95% CI)	*p* value	OR (95% CI)	*p* value	OR (95% CI)	*p* value
Primary endpoint						
NYHA ≥III	5.91 (2.02–17.30)	0.001	6.02 (1.33–18.77)	0.002	6.30 (2.04–19.67)	0.001
Rescue CRT	0.49 (0.40–0.60)	0.01				
CKD	0.41 (0.16–1.03)	0.06				
Loop diuretics	0.32 (0.09–1.11)	0.06				
Anticoagulation	0.51 (0.23–1.15)	0.10				
Atrial fibrillation	0.51 (0.23–1.15)	0.10				
ACEI-ARB	2.07 (0.84–5.11)	0.11	2.55 (0.91–7.11))	0.07	2.38 (0.87–6.49)	0.09
Surgery duration	1.87 (0.85–4.11)	0.12				
QRS = 130–150 ms	0.50 (0.20–1.27)	0.14				
One-year mortality						
Rescue CRT	58.8 (6.55–528-28)	< 0.001	44.67 (2.91–686)	0.01	63.28 (4.47–896)	0.002
CRT-D	0.23 (0.07–0.75)	0.01				
Atrial fibrillation	4.03 (1.07–15.20)	0.03	6.72 (0.99–45.61)	0.04		
QRS > 150 ms	2.64 (0.86–8.15)	0.08	3.79 (0.79–18.21)	0.10		
Surgery duration	0.39 (0.13–1.2)	0.09	0.11 (0.02–0.63)	0.01	0.14 (0.03–0.76)	0.02
CKD	3.54 (0.75–16.67)	0.09				
Upgrade	0.36 (0.08–1.69)	0.10				
Anticoagulation	2.43 (0.72–8.13)	0.14				
Beta-blockers	0.42 (0.12–1.42)	0.15				
Ivabradine	2.63 (0.60–11.41)	0.18	9.99 (1.17–85.42)	0.04	6.36 (1.10–36.90)	0.04
ACEI-ARB	0.47 (0.15–1.49)	0.19				

Anticoagulation and atrial fibrillation were significantly correlated in the ≥75-year-old group (Pearson coefficient 0.79, *p* < 0.001). We performed two different regression models if the *p* values were both < 0.2 in the univariate analysis. We reported here variables with *p* values ≤0.2 in the univariate analysis and ≤ 0.10 in the multivariate analysis. ACEI: angiotensin-converting enzyme inhibitor; ARB: angiotensin II receptor blocker; CKD: chronic kidney disease (defined by estimated glomerular filtration < 60 ml/mn/1.73 m^2^); CRT: cardiac resynchronization therapy; CRT-D: CRT with defibrillator; NYHA: New York Heart Association

**Table 3:** Univariate and multivariate analyses of outcomes in the < 75-year-old group

	Univariate analysis		Multivariate analysis			
Primary endpoint	OR (95% CI)	*p* value	OR (95% CI)		*p* value	
Atrial fibrillation	0.36 (0.18–0.71)	0.003	0.28 (0.13–0.62)		0.001	
CRT-D	2.39 (0.85–6.70)	0.09				
Male sex	2.39 (0.85–6.70)	0.09				
CKD	0.53 (0.26–1.07)	0.08				
Ivabradine	2.25 (0.85–5.97)	0.10				
NYHA ≥III	1.74 (0.89–3.41)	0.11	2.09 (0.95–4.59)		0.07	
QRS < 130 ms	0.51 (0.21–1.24)	0.13				
QRS > 150 ms	1.63 (0.83–3.17)	0.15				
	Univariate analysis		Multivariate analysis (model 1 with atrial fibrillation)		Multivariate analysis (model 2 with anticoagulation)	
One-year mortality	OR (95% CI)	*p* value	OR (95% CI)	*p* value	OR (95% CI)	*p* value
Atrial fibrillation	23.68 (3.04–184.30)	< 0.001	14.35 (1.6–125.90)	0.02		
Rescue CRT	17.48 (4.97–61.44)	< 0.001	14.32 (2.61–79.20)	0.002	15.81 (3.35–75.10)	< 0.001
CRT-D	0.11 (0.04–0.34)	< 0.001				
Beta-blockers	0.16 (0.05–0.51)	0.001				
CKD	5.93 (1.61–21.83)	0.003	5.96 (1.13–31.30)	0.04	7.65 (1.47–39.61)	0.02
Anticoagulation	7.71 (1.68–33.26)	0.003			5.32 (0.96–29.40)	0.06
ACEI-ARB	0.26 (0.09–0.78)	0.01				
Diabetes mellitus	2.86 (1.02–8.01)	0.04				
Loop diuretics	5.67 (0.72–44.56)	0.07				
NYHA ≥III	4.24 (1.16–15-48)	0.02				
QRS > 150 ms	0.46 (0.15–1.38)	0.16				
Complications	2.06 (0.69–6.08)	0.19	4.20 (0.91–18.91)	0.07		

Anticoagulation and atrial fibrillation were significantly correlated in the < 75-year-old group (Pearson coefficient 0.69, *p* < 0.001). We performed two different regression models if the p values were both < 0.2 in the univariate analysis. We reported here variables with *p* values ≤0.2 in the univariate analysis and ≤ 0.10 in the multivariate analysis. ACEI: angiotensin-converting enzyme inhibitor; ARB: angiotensin II receptor blocker; CKD: chronic kidney disease (defined by estimated glomerular filtration < 60 ml/mn/1.73 m^2^); CRT: cardiac resynchronization therapy; CRT-D: CRT with defibrillator; NYHA: New York Heart Association

### Secondary endpoints

#### All-cause mortality

**Fig. 1:**
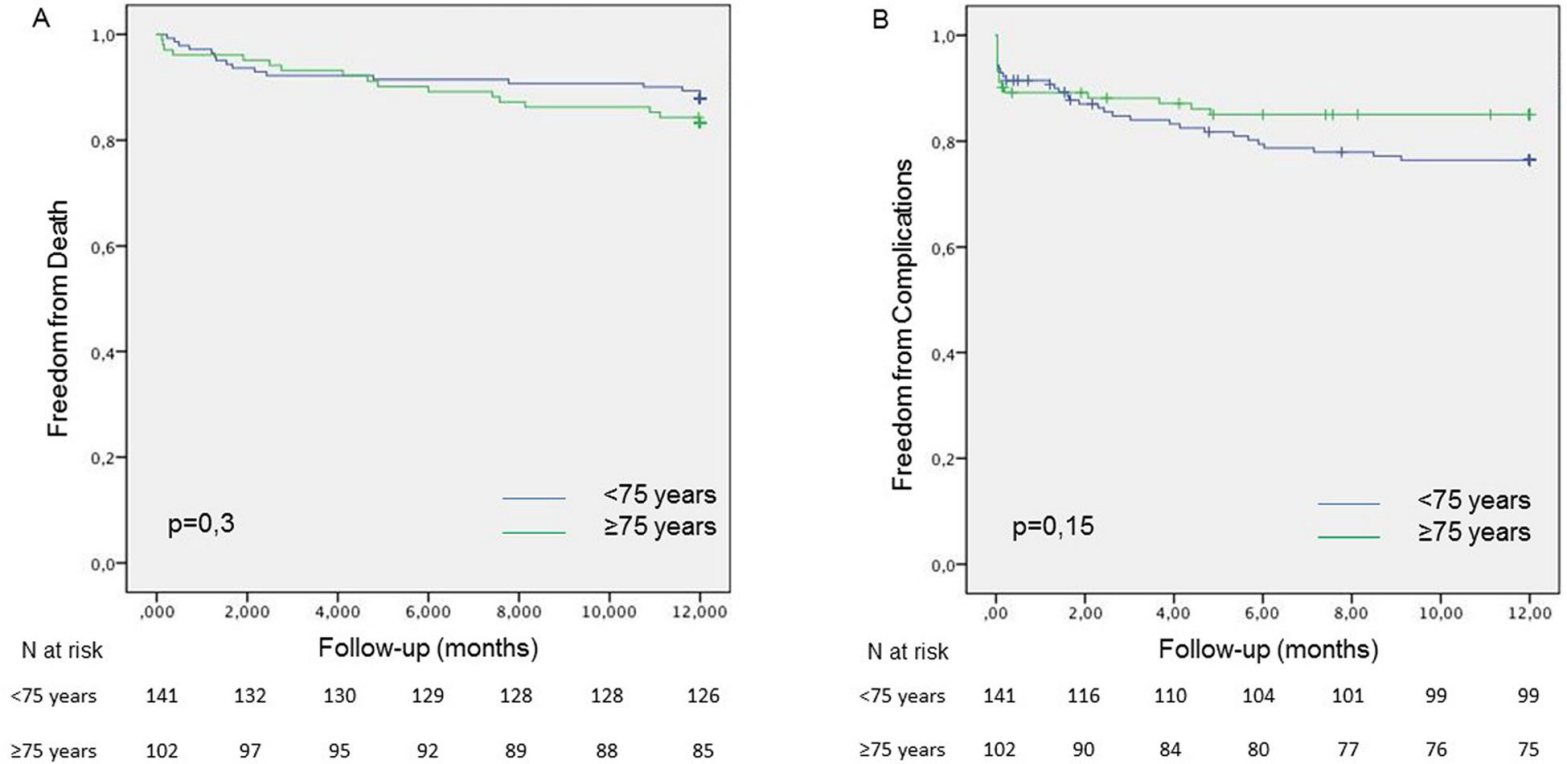
Kaplan-Meier curves for one-year mortality and complications in the ≥75- and < 75-year-old groups. Panel **a**: Overall survival, Panel **b**: Complications. The ≥75-year-old group corresponds to green, and the < 75-year-old group corresponds to blue. Comparisons were made with the log-rank test

At one year, the mortality rates were 12 and 17% in the < 75- and ≥ 75-year-old groups, respectively (*p* = 0.31). The survival curves are represented in Fig. 1. Defibrillator use was associated with better survival in the univariate analysis (*p* = 0.01) but not after the logistic regression. The multivariate analysis showed that rescue CRT (*p* = 0.01 and 0.002 in the ≥75- and < 75-year-old groups, respectively) and AF (*p* = 0.04 and 0.02 in the ≥75- and < 75-year-old groups, respectively) were associated with mortality in both groups. CKD was predictive of one-year mortality only in the < 75-year-old group (*p* = 0.04), whereas ivabradine use was associated with mortality only in the ≥75-year-old group (*p* = 0.04).

#### Complications

Eighty-two complications were reported in 45 patients (19%) in the study population. There was no difference in the one-year complication rates between age groups, which were 15 and 21% in the ≥75- and < 75-year-old groups, respectively (*p* = 0.19). Lead dislodgment and reinterventions were the most frequent issues, with a trend towards a greater rate of reinterventions in the < 75-year-old group (*p* = 0.07). Only defibrillator use (*p* = 0.003) and QRS duration (*p* = 0.02) were found to be independent predictive factors in the overall population. There was no age-related predictor of complications.

#### Device therapies in patients with a defibrillator (*n* = 175)

Nine (17%) patients ≥75 years old received appropriate therapies, whereas 11 (9%) patients < 75 years old received appropriate therapies (*p* = 0.2). Only four patients, who were all in the < 75-year-old group, received inappropriate therapies.

## Discussion

In our study, older patients who underwent CRT presented more comorbidities than did their younger counterparts. Such differences have been highlighted in other studies [8] and registries [7, 15]. However, they tended to have a response to CRT similar to that of younger individuals, considering a clinical combined endpoint for CRT effectiveness defined as survival for one year with no heart failure hospitalization and improvement by ≥ one NYHA class. Patients ≥75 years old with a previous diagnosis of a NYHA class ≥III were found to respond better than less symptomatic individuals, whereas functional status was not predictive factor for CRT response in younger individuals. CRT was also found to be safe in both age groups, with no difference in the mortality rate or in the complication rate. The strongest predictors of mortality, rescue CRT and AF, were not age-related.

Whether CRT can be as efficient in older patients as it is in younger patients remains unknown. Observational studies have shown that older patients are likely to respond to CRT as well as younger patients are [8, 9, 16, 17]. In our study, age was not predictive factor for CRT response, as a subanalysis of two randomized trials comparing three age groups (< 65; 65–75; > 75 years) reported the same improvements in LVEF and NYHA class regardless of age [18]. In contrast, Maass et al. recently identified age < 60 years as a predictive factor for reverse ventricular remodeling after CRT [19]. Little is known about response predictors according to age. We reported here for the first time that NYHA class ≥III at the time of implantation was associated with CRT response in the ≥75-year-old individuals but not in younger patients. Conversely, a recent study showed that NYHA class ≤III was an independent predictor of CRT benefit in a mid-sixties population [20]. In contrast, AF has been associated with poor results of CRT [21]. In our study, this negative impact of AF was found in the younger group, whereas patients ≥75 years with AF improved as well as those in sinus rhythm did.

There are conflicting results regarding the impact of CRT on mortality among older patients. We found no difference in mortality rates between age groups. This result was also reported in previous studies [9, 10]. Killu et al. showed, after performing a multivariate analysis, that there was no significant difference in survival between patients aged ≤80 and > 80 [10]. In contrast, several authors noted higher mortality rates among older patients [11, 15, 17, 22] but more noncardiac deaths in these patients [22] and a similar time to death between age groups [11]. Diabetes mellitus, CKD and low functional capacity were predictive of worse survival in ≥75-year-oldpatients during a long-term follow-up period [22]. In our study, we found that one-year mortality was strongly associated with rescue CRT and AF, which were not age-related. CKD was not associated with mortality in the ≥75 age group and was only associated with mortality in younger patients. The COMPANION trial reported that CRT reduced all-cause mortality only in patients with a defibrillator [2], whereas defibrillator use was not associated with survival in either the < 75- or ≥ 75-year-old patients in our study. We also found that ivabradine use was predictive of deaths in older patients. We have no explanation for this finding, as it has not been reported before and may be hazard-related. In contrast, a recent study showed that ivabradine was well tolerated in patients ≥70 years old with systolic HF [23].

There was no difference concerning complications after CRT implantation between age groups. Despite higher rates of comorbidities and advanced HF, age ≥ 75 years was not associated with a higher complication rate. This result is consistent with those in most published data [9, 15]. Höke et al. reported only a trend towards a higher incidence of pneumothorax and pocket hematoma in ≥75-year-old patients [22]. Nevertheless, the risk of adverse events is the most frequently reported explanation for the CRT age limit observed in European centers [24]. As reported recently, we found that CRT defibrillator use was more strongly associated with complications than was CRT pacemaker use, regardless of age [25].

Thus, our results suggest that CRT is essential for older adults. It is difficult to “optimize” treatment for these patients because CKD and hypotension limit the medical therapies, disabilities limit cardiac rehabilitation and other age-related features defining frailty in elderly individuals limit therapies. Patients over 75 years of age with advanced HF and a high NYHA class, even with AF, should be considered for CRT. Age should not be a limitation itself; rather, individual risk has to be evaluated in combination with other comorbidities to select CRT candidates and whether this device is associated with defibrillator use.

### Limitations

Some limitations of our study should be addressed. This retrospective study with a small sample size may be underpowered and is subject to selection bias. Except in cases of rescue CRT, we can assume that the frailest elderly patients did not undergo implantation, which could lead to an overestimate of the CRT benefit among the ≥75-year-old group. We did not report other data about functional status like the six-minute walking distance. Other studies reported similar improvements in the six-minute walking distance after CRT in older patients compared to younger counterparts [8, 9]. Interestingly, the six-minute walking distance was found predictive of mortality in elderly patients undergoing cardiac rehabilitation after coronary bypass grafting, whereas LVEF was not [26]. Causes of death were not adjudicated and therefore were not analyzed. Last, we did not retrieve cognitive status, quality of life scores and end-of-life experiences, but these outcomes are important, especially for older patients [6]. Additional studies should be designed to address these issues, and test other potential predictors of clinical outcomes between younger and older patients with CRT.

## Conclusion

Our study showed that very symptomatic older adults are likely to respond to CRT and that AF was not associated with worse outcomes. We also highlighted the safety and efficiency of CRT regardless of patient age.

## Data Availability

The datasets used and/or analyzed during the current study are available from the corresponding author upon reasonable request.

## Abbreviations

AF: Atrial fibrillation
CKD: Chronic kidney disease
CRT: Cardiac resynchronization therapy
HF: Heart failure
LBBB: Left-bundle branch block
LVEF: Left ventricular ejection fraction
NYHA: New York Heart Association
